# Two different evolutionary lines of filamentous phages in *Ralstonia solanacearum*: their effects on bacterial virulence

**DOI:** 10.3389/fgene.2015.00217

**Published:** 2015-06-18

**Authors:** Ahmed Askora, Takashi Yamada

**Affiliations:** ^1^Department of Microbiology and Botany, Faculty of Science, Zagazig University, ZagazigEgypt; ^2^Department of Molecular Biotechnology, Graduate School of Advanced Sciences of Matter, Hiroshima University, Higashi-HiroshimaJapan

**Keywords:** filamentous phage, integration, pathogenic bacteria, virulence change

## Abstract

The integration and excision of various filamentous phage genomes into and out of their host chromosomes occurs by site-specific recombination. The mechanisms proposed for these events include reactions mediated by phage-encoded recombinases and host recombination systems. Site-specific integration of filamentous phages plays a vital role in a variety of biological functions of the host, such as phase variation of certain pathogenic bacterial virulence factors. The importance of these filamentous phages in bacterial evolution is rapidly increasing with the discovery of new phages that are involved in pathogenicity. Studies of the diversity of two different filamentous phages infecting the phytopathogen *Ralstonia solanacearum* provide us with novel insights into the dynamics of phage genomes, biological roles of prophages, and the regulation and importance of phage–host interactions.

## Filamentous Phages and Pathogenic Bacteria

**FIGURE 1:**
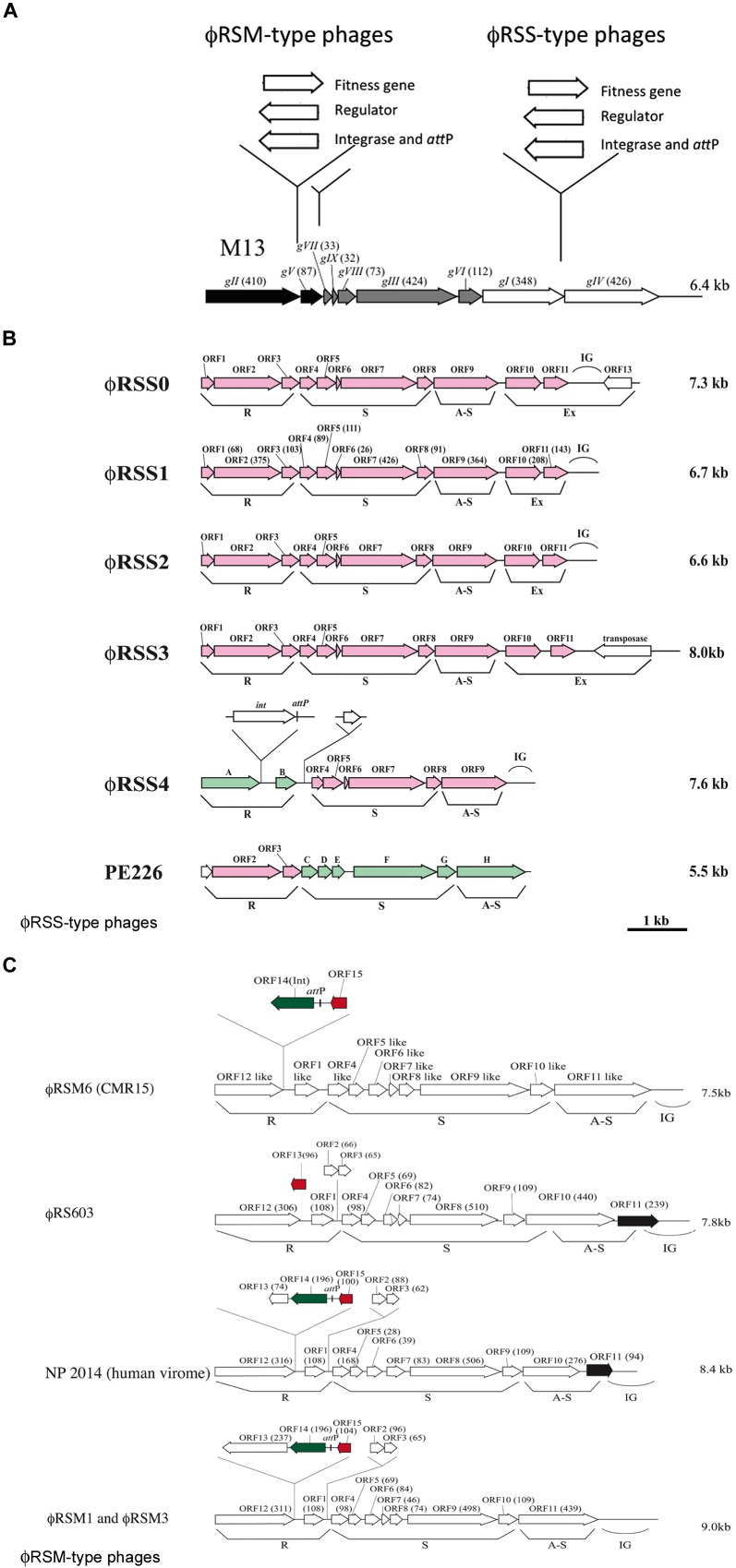
**Diversity of genomic arrangement in filamentous phages of *Ralstonia solanacearum***. **(A)** For ϕRSM-type and ϕRSS-type phages, gene insertion sites are shown along the linear genomic map of *Escherichia coli* phage M13 (Model and Russel, 1988; Marvin, 1998). Arrows indicate the direction of transcription and represent open reading frames (ORFs) or genes. The functional modules for replication (R), structure (S), and assembly and secretion (A-S) are indicated according to the M13 model. ORF sizes (in amino acids) are in parentheses. IG, intergenic region. **(B)** Genomic organization of ϕRSS-type phages. According to the *E. co*li M13-model, ORFs identified in the phage genome are grouped into the R, S, and AS functional modules. IG, large intergenic region. ϕRSS0, ϕRSS2, ϕRSS3, and ϕRSS4 were derived from prophages of strains C319, M4S, MAFF106611, and MAFF211271, respectively. PE226 is a phage of Korean strains of *R. solanacearum* (Murugaiyan et al., 2010). ORFs shown in pink are homologous to ϕRSS1 ORFs, and those in green are homologous to ϕRSM-type ORFs. **(C)** Genomic organization of ϕRSM-type phages. ϕRSM1 and ϕRS603 were isolated from soil (Kawasaki et al., 2007; Bich Van et al., 2014). ϕRSM3 and ϕRSM6 are prophages of strains MAFF730139 and CMR15 (phylotype III, Remenant et al., 2010), respectively. NP204 is similar to a phage found in the human virome (*Ralstonia* phage 1 NP2014, accession no. AHI87735.1). ORFs shown in green, red, and black are genes encoding an integrase (Int), transcriptional repressor, and ϕRSS1-*ORF11*-like ORF, respectively.

Bacteriophages of the genus *Inovirus* are filamentous particles containing a circular single-stranded (ss) DNA genome. This kind of phage does not lyse host cells, but it establishes a persistent association with the host, producing and releasing phage particles from the growing and dividing host cells. The genome of inoviruses, represented by the *Escherichia coli* F-pilus-specific phage Ff (f1, fd, or M13), is generally organized in a modular structure in which functionally related genes are grouped together (Horiuchi, 1997; Rakonjac et al., 2011; Mai-Prochnow et al., 2015). Three functional modules are always present: the replication module (R), the structural module (S), and the assembly and secretion module (A-S; **Figure 1A**). The R module contains the genes encoding rolling-circle DNA replication and ssDNA-binding proteins pII, pV, and pX (Horiuchi, 1997). The S module contains genes for the major (pVIII) and minor coat proteins (pIII, pVI, pVII, and pIX). The gene *gIII* encodes the host recognition or adsorption protein pIII (Wang et al., 2006). The A-S module contains the genes for morphogenesis and extrusion of the phage particles (*gI* and *gIV*; Marvin, 1998). The gene *gIV* encodes protein pIV, an aqueous channel (secretin) in the outer membrane, through which phage particles exit from the host cells (Marciano et al., 1999). Although some phages encode their own secretins, others use host products (Davis et al., 2000). For the general infection cycle of inoviruses, see recent reviews (Rakonjac et al., 2011; Mai-Prochnow et al., 2015).

In pathogenic bacteria of either animals or plants, filamentous phage infection has been demonstrated to affect virulence. Examples include (i) enhancing production of virulence factors such as extracellular polysaccharides (EPSs) in Xf- or Lf-infected *Xanthomonas campestris* (Kamiunten and Wakimoto, 1982; Tseng et al., 1990), (ii) induction of biofilm formation in Pf4-producing *Pseudomonas aeruginosa* (Webb et al., 2004; Rice et al., 2009), and (iii) reduced twitching motility in ϕRSM-infected *Ralstonia solanacearum* (Addy et al., 2012a) and in XacF1-infected *X. citri* (Ahmad et al., 2014). These are likely caused by changes in the host cell surface where phage proteins are secreted and filamentous particles are assembled. More direct involvement of filamentous phages in host virulence is well characterized in *Vibrio cholerae*. The pathogenicity of this severe diarrheal disease–causing bacterium depends on two key virulence factors, the toxin co-regulated pilus and cholera toxin. Cholera toxin genes are encoded on the filamentous phage CTXϕ and are introduced into bacterial cells by phage integration mediated by the host *dif*/XerCD recombinase system (Huber and Waldor, 2002; Davis and Waldor, 2003). Also, the filamentous prophage MDA was found at multiple sites in the host chromosome associated with invasive isolates of *Neisseria meningitidis* (Bille et al., 2005). The prophage Ypfϕ was reported to contribute to the pathogenicity of the plague bacillus, *Yersinia pestis* (Derbise et al., 2007). The acquisition of the filamentous phage CUS-1 encoding *puvA* was thought to contribute to the expression of a high-virulence phenotype in *Escherichia coli* O18:K1:H7 (Gonzalez and Allen, 2003). In these cases, filamentous phages with genes encoding toxins, virulence-enhancing factors, or host fitness factors were integrated into the host genome by various mechanisms. For other examples of filamentous phages infecting pathogenic bacteria, see the recent review by Ilyina (2015).

## Different Strategies for Filamentous Phage DNA Integration into the Host Genome

To date, four different integration mechanisms used by filamentous phages have been described (**Table 1**). Well-characterized filamentous coliphages, such as M13 and fd, typically do not take a lysogenic replication cycle and replicate exclusively as an episome in their host bacteria (Model and Russel, 1988; Rakonjac et al., 2011). Some filamentous phages, including CTXϕ of *V. cholerae*, accomplish site-specific integration into the *dif* site of the bacterial chromosome by using the host XerC/D recombination system (Huber and Waldor, 2002). Filamentous phages such as VEJϕ of *Vibrio parahaemolyticus* (Campos et al., 2010); Cf1c, Cf1t, Cf16v1, and ϕLf of *X. campestris* (Campos et al., 2003); Xf1c and XacF1 of *X. citri* (Ahmad et al., 2014); Xfϕf1 of *Xylella fastidiosa* (de Mello Varani et al., 2008); and Ypfϕ of *Yersinia pestis* (Lesterlin et al., 2004) also seem to use the host XerC/D recombination for their integration. In contrast, ϕRSM1 and ϕRSM3 of *R. solanacearum* encode a site-specific integrase (Int) of the resolvase/invertase subfamily of serine recombinases (Askora et al., 2011). This kind of serine recombinase mediates recombination involving the process of double-strand breakage followed by rotation and religation. Both integrative and excisive recombination reactions were catalyzed by ϕRSM-Int (Askora et al., 2011). The phage *att*P corresponded to the 13 b sequence at the 5^′^ of serine tRNA (UCG) of the host. The same unit of integration (Int–*att*P) was also found in a *R. pickettii* 12J filamentous prophage and in *Burkholderia pseudomallei* 668 prophage. A different strategy to integrate DNA into the host genome by filamentous phages may be via transposases. Kawai et al. (2005) observed filamentous prophages integrated into the chromosome of *Neisseria* species. Each prophage copy of the neisserial filamentous phage (*Nf*) was flanked by a duplication of the 5^′^-CT and carried an open reading frame (ORF) encoding a transposase homolog (pivNM/irg), suggesting the transposase-mediated integration of *Nf* DNA into host bacterial chromosomes. Bille et al. (2005) actually showed that the integration of *Nf* DNA is mediated by its own transposases (pivNM/irg). Meanwhile, Webb et al. (2004) and Mooij et al. (2007) characterized two filamentous prophages, Pf4 and Pf5, in the genome of *P. aeruginosa* PAO1 and PA14, respectively. Both prophages were integrated into tRNA genes of their host, probably in a reaction mediated by their own Int from the tyrosine-recombinase family. Thus, at least four different strategies for the integration of filamentous bacteriophage DNA into the host chromosome are known (Askora et al., 2012; **Table 1**).

**Table 1:** Comparison of site-specific recombination systems in filamentous phages.

Phage	Recombinase	Target sequence *attP*	Host	Reference
CTXϕ	XerC/XerD	*dif*	*Vibrio cholerae*	Huber and Waldor (2002)
ϕRSM	Resolvase/Invertase	Ser tRNA (3′-13 bp)	*Ralstonia solanacearum*	Askora et al. (2009)
Nf	Transposase	20-bp inverted repeat (dRS3)	*Neisseria* sp.	Kawai et al. (2005)
Pf4	Tyrosine recombinase	Gly tRNA (3′-27 bp)	*Pseudomonas aeruginosa*	Webb et al. (2004)
ϕRSS1	XerC/XerD	*dif*	*R. solanacearum*	Yamada (2013)
XacF1	XerC/XerD	*dif*	*Xanthomonas campestris*	Ahmad et al. (2014)


## Structural and Biological Diversity of Two Different Filamentous Phages Infecting *Ralstonia solanacearum*

*Ralstonia solanacearum* is a Gram-negative β-proteobacterium that causes bacterial wilt disease in many important crops including tomato, potato, tobacco, eggplant, banana, ginger, and mulberry. Because of its wide geographic distribution and unusually broad host range (more than 50 plant families), it is responsible for significant crop losses worldwide (Hayward, 2000; Denny, 2006). Filamentous phages that were found to infect strains of *R. solanacearum* were classified into two groups, ϕRSS-type and ϕRSM-type phages. ϕRSS1 is a representative of ϕRSS-type phages and is a relatively small particle (1.1 μm in length) containing an ssDNA genome of 6,662 nt (with a GC content of 62.6%) encoding 11 ORFs (Kawasaki et al., 2007). Genomic DNA of these types of phage was frequently found integrated in the host genome; 23 of 24 strains tested (all isolated in Japan) showed positive hybridization signals in Southern blot analysis (Yamada et al., 2007). Some prophage sequences were determined (**Figure 1B**). ϕRSS0, ϕRSS2, and ϕRSS3 were derived from prophages of strain C319, M4S, and MAFF106611, respectively (Yamada, 2012). Compared with the M13 gene organization, additional genes are inserted within or next to the A-S module in these ϕRSS genomes (**Figure 1A**). In the case of ϕRSS0, a putative regulatory gene (with similarity to transcriptional repressors; *ORF13*) is inserted in the reverse orientation with two unknown ORFs (*ORF10* and *ORF11*). There is an *R. solanacearum* *dif* sequence within *ORF13* that serves as an *att*P site for integration into the host genome by host XerC/D recombinases (Yamada, 2013). In the case of ϕRSS3, an additional gene encoding a transposase (IS4 family) was located in the reverse orientation. This may function for integration of the phage DNA in some occasions like the *Neisseria* cases described above (Kawai et al., 2005). Therefore, these ϕRSS variations represent the possibility of functional equipment at this genomic region with genes for host fitness, integration, and regulatory functions (**Figure 1A**).

Another type of filamentous phage of *R. solanacearum* revealed a different story of evolution. ϕRSM1, the first phage to be classified as a member of the ϕRSM-type phages is a longer filamentous particle (1.5 μm in length) containing ssDNA of 9,004 nt (with a GC content of 59.9%) as the genome (Kawasaki et al., 2007; Yamada et al., 2007). A total of 15 ORFs were found on the ϕRSM1 genome including five extra genes in addition to M13-core genes. The extra genes are inserted within the R module or between the R and S modules (**Figure 1C**). Two of these extra genes (*orf14* and *orf15*) encode a DNA resolvase/invertase-like serine recombinase functioning as an Int (Askora et al., 2011) and a transcriptional repressor (Addy et al., 2012a), respectively. There was an *att*P site between *orf14* and *orf15* (**Figure 1C**). The function of the other extra genes is not known. In contrast to ϕRSS phages, the integration of ϕRSM-type phage DNA into the genome of strains isolated in Japan was not frequent; 6 of 24 strains tested showed positive signals in genomic Southern blot analysis. However, genomic sequences of *R. solanacearum* strains and related β-proteobacteria in the databases frequently showed ϕRSM-like prophage sequences. A comparison of those sequences revealed the genomic diversity of ϕRSM-type phages as shown in **Figure 1C** and Supplementary Figure S1. Only one gene encoding a putative repressor (corresponding to ϕRSM1 *ORF15*) is located within the extra region in the R module of *R. solanacearum* phage ϕRS603 (Bich Van et al., 2014), whereas ϕRSM6 in strain CMR15 (phylotype III) contained an Int gene (*ORF14*) in addition to the repressor gene (*ORF15*; Askora et al., 2014). Like ϕRSM1, ϕRSM3, ϕRSM4 in strain UW551 (phylotype II), ϕRSM5 in strain IPO1609 (phylotype II), and ϕRSM7 in strain Y45 (phylotype IB) contained three genes within this region (*ORF13*, *ORF14*, and *ORF15*) with the same organization (**Figure 1C**). However, it is noteworthy that there are two different regulatory systems, where the amino acid sequence of ORF15 and its upstream regulatory nucleotide sequence are different in phages infecting different phylotypes (Askora et al., 2014). ϕRSM1, ϕRSM3, and Y45, which infect strains of phylotype I, share similar regulatory systems, whereas ϕRSM5, ϕRSM6, and ϕRSM7, which infect strains of phylotypes II or III, contained another system. Very similar ϕRSM sequences were also found in the genomes of *R. syzgii* and *R. pickettii* (Askora et al., 2014). This kind of phage may have an extensive host range in b-proteobacteria. Interestingly, a ϕRSM homolog was found in the human virome (*Ralstonia* phage 1 NP2014, accession no. AHI87735.1) as shown in **Figure 1C**. *Ralstonia* phage 1 NP2014 possesses a circular ssDNA genome that is highly homologous to those of ϕRSM1 and ϕRSM3. *Ralstonia* phage 1 NP2014 contains a unique *ORF11* with high similarity to ϕRSS0 *ORF11* (**Figure 1B**).

As described above, two groups of filamentous phages of *R. solanacearum* have used different mechanisms for the evolution of genomic arrangements (**Figure 1A**). However, there may have been some opportunities for them to infect the same host cells by chance, which would have made it possible for the two types of phage to hybridize. Actually, such forms were detected (**Figure 1B**). A prophage (ϕRSM4) found in strain MAFF211271 showed a gene arrangement with the ϕRSM-type R module containing genes for an Int and regulator and with ϕRSS-type S and A-S modules (Yamada, 2012). A smaller filamentous phage, PE226, was isolated with Korean strains and showed a gene arrangement with a ϕRSS-type R module and ϕRSM-type S and A-S modules (Murugaiyan et al., 2010). Therefore, further genomic diversity by mixing these two types of phage gene arrangement is not surprising.

## Filamentous Phage Diversity and Effects on the Host Virulence and Evolution in *R. solanacearum*

Both ϕRSS-type and ϕRSM-type filamentous phages affect the host physiology including virulence. ϕRSS1-infected cells showed enhanced virulence on tobacco (Yamada et al., 2007) and tomato plants (Addy et al., 2012b). The virulence-enhancing effects by ϕRSS1 infection can be explained as follows: surface-associated ϕRSS1 particles (or phage proteins) may change the surface nature (hydrophobicity) of host cells to generate a high local cell density, resulting in early activation of *phcA*, the global virulence regulator, or lack of *orf13*, which is absent from the ϕRSS1 genome (Addy et al., 2012b). The reduced virulence observed for ϕRSS0-infected cells may be caused by the function(s) of ORF13 encoded by ϕRSS0 (Yamada, 2013). Contrasting to the ϕRSS1 effects, upon infection by ϕRSM phages, the host cells showed loss of virulence phenotypes (Addy et al., 2012a). This loss of virulence effect of ϕRSM infection can be explained in three ways: (i) reduced twitching motility and reduced amounts of type IV pili (Tfp), (ii) lower levels of β-1,4-endoglucanase (Egl) activity and EPS production, and (iii) reduced expression of certain virulence/pathogenicity genes (*egl*, *pehC*, *phcA*, *phcB*, *pilT,* and *hrpB*) in the infected cells (Addy et al., 2012a).

Thus, phages sometimes help host bacteria infect plants by enhancing bacterial virulence, and they sometimes interrupt bacterial infection of plants by repressing host genes involved in virulence. Such contradictory effects of these phages largely depend on the phage state, for example, replicating freely in the host, existing as a stable prophage (with Int), or expressing a special transcriptional regulator (Yamada, 2013). In general, the phage-encoded regulator somehow affects the expression of host genes involved in virulence, mostly through repression, in both ϕRSS-type and ϕRSM-type phages. However, integration into the host genome may cause a change in the regulatory function (namely direct effects on the host gene expression may be relaxed). As described above, cell surface changes caused by filamentous phage secretion affect quorum sensing, twitching motility, and biofilm formation. Depending on the lifestyle of host bacterial cells in the environment, phage effects are different, and an advantageous state of cells with phage will be selected under the conditions. Cells whose virulence is enhanced by phage will predominate in the pathogenic stage. Similar types of phage involvement in host virulence regulation may be universal because ϕRSS- or ϕRSM-related sequences are frequently found in various bacterial genomic sequences, including *R. picketti* (accession no. CP001645), *R. syzygii* (FR854090), *Burkholderia rhizoxinica* (FR687359), *Pectobacterium wasabiae* (CP001790), and *Erwinia carotovora* (BX950851). The diversity observed in the genome arrangement and biological effects of filamentous phages infecting the phytopathogen *R. solanacearum* will serve as a good reference to consider interactions between various pathogenic bacteria and their phages.

### Hypothesis

Filamentous phages are widely disseminated and exist as prophage states in different strains of pathogenic bacteria. They might evolve rapidly and play roles in the introduction of new genes into their hosts. Therefore, it is highly likely that filamentous phages are mediating the ecological adaptation and virulence of their hosts and thus play significant roles in the evolution of bacterial species.

## Supplementary Material

The Supplementary Material for this article can be found online at: http://journal.frontiersin.org/article/10.3389/fgene.2015.00217

## Conflict of Interest Statement

The authors declare that the research was conducted in the absence of any commercial or financial relationships that could be construed as a potential conflict of interest.
