# Supplementary material for: Two different evolutionary lines of filamentous phages in Ralstonia solanacearum: their effects on bacterial virulence
Source: Front Genet. 2015 Jun 18;6:217. doi: 10.3389/fgene.2015.00217 (PMC4471427; doi:10.3389/fgene.2015.00217)
Supplement: Supplementary file 1 [file Presentation_1.PPTX]

## Slide 1
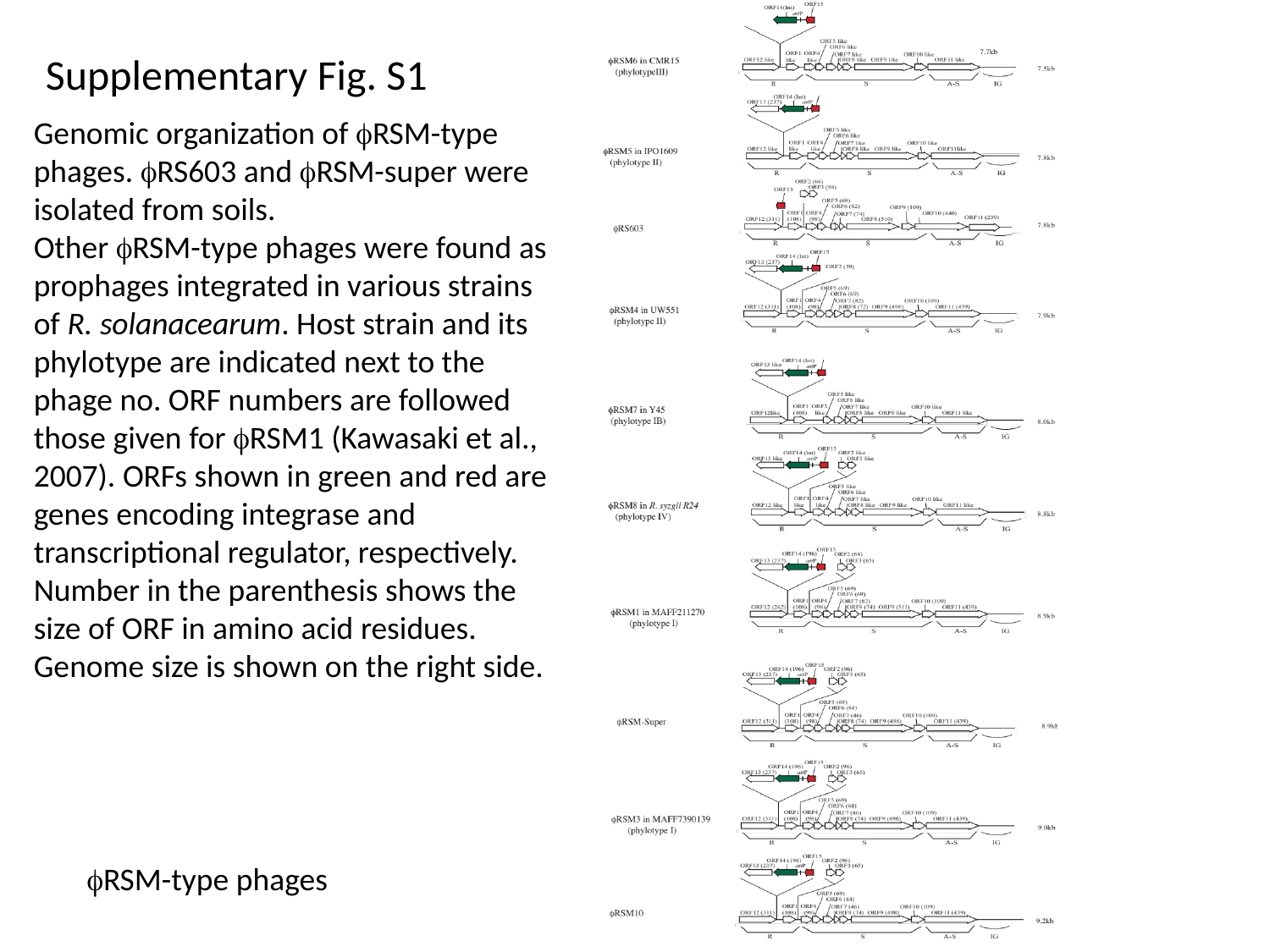

Supplementary Fig. S1
Genomic organization of fRSM-type phages. fRS603 and fRSM-super were isolated from soils.
Other fRSM-type phages were found as prophages integrated in various strains of R. solanacearum. Host strain and its phylotype are indicated next to the phage no. ORF numbers are followed those given for fRSM1 (Kawasaki et al., 2007). ORFs shown in green and red are genes encoding integrase and transcriptional regulator, respectively.
Number in the parenthesis shows the size of ORF in amino acid residues.
Genome size is shown on the right side.
fRSM-type phages
